# A Non-Vacuum Coating Process That Fully Achieves Technical Goals of Bipolar Plates via Synergistic Control of Multiple Layer-by-Layer Strategy

**DOI:** 10.3390/molecules30122543

**Published:** 2025-06-11

**Authors:** Qiaoling Liu, Xiaole Chen, Menghan Wu, Weihao Wang, Yinru Lin, Zilong Chen, Shuhan Yang, Yuhui Zheng, Qianming Wang

**Affiliations:** Key Laboratory of Analytical Chemistry for Biomedicine, School of Chemistry, Guangzhou South China Normal University, Guangzhou 510006, China; 2021022639@m.scnu.edu.cn (Q.L.); 2024022855@m.scnu.edu.cn (X.C.); 2022022616@m.scnu.edu.cn (M.W.); 2023022500@m.scnu.edu.cn (W.W.); 20222421031@m.scnu.edu.cn (Y.L.); hychen7788@126.com (Z.C.); skinfade101@gmail.com (S.Y.)

**Keywords:** composite coating, corrosion resistance, electrochemical analysis, non-vacuum strategy

## Abstract

The primary challenge associated with stainless steel in fuel cell operation is its susceptibility to corrosion, which leads to increased contact resistance and subsequent degradation of electrochemical performance. In general, the protective layers have been loaded onto the metal surface by widely used traditional techniques such as physical vapor deposition (PVD), or cathode arc ion plating. However, the above sputtering and evaporation ways require a high-vacuum condition, complicated experimental setups, higher costs, and an elevated temperature. Therefore, herein the achievement for uniform coatings over a large surface area has been realized by using a cost-effective strategy through a complete wet chemical process. The synergistic regulation of two conductive components and a plastic additive has been employed together with the entrapment of a surfactant to optimize the microstructure of the coating surface. The assembly of layered graphite and a polystyrene sphere could maintain both the high corrosion resistance feature and excellent electrical conductivity. In particular, the intrinsic vacant space in the above physical barriers has been filled with fine powders of indium tin oxide (ITO) due to its small size, and the interconnected conductive network with vertical/horizontal directions would be formed. All the key technical targets based on the U.S. Department of Energy (DOE) have been achieved under the simulated operating environments of a proton exchange membrane fuel cell. The corrosion current density has been measured as low as 0.52 μA/cm^2^ (for the sample of graphite/mixed layer) over the applied potentials from −0.6 V to 1.2 V and its protective efficiency is evaluated to be 99.8%. The interfacial contact resistance between the sample and the carbon paper is much less than 10 mΩ·cm^2^ (3.4 mΩ·cm^2^) under a contact pressure of 165 N/cm^2^. The wettability has been investigated and its contact angle has been evolved from 48° (uncoated sample) to even 110°, providing superior hydrophobicity to prevent water penetration. Such an innovative approach opens up new possibilities for improving the durability and reducing the costs of carbon-based coatings.

## 1. Introduction

Proton exchange membrane fuel cells (PEMFCs), which serve as the power source for fuel cell vehicles, directly convert the chemical energy of hydrogen into electrical energy, with only water as the byproduct [1]. This technology offers high energy conversion efficiency and zero emissions, making it widely regarded as a key component of future clean energy systems. As the most indispensable structure in fuel cells, the bipolar plate plays a significant role in facilitating electrochemical reactions and effectively distributing reactants [2]. Over the years, various types of bipolar plates have been developed to optimize the performance and durability of fuel cells [3].

In the field of raw materials, stainless steel bipolar plates have generated considerable interest due to their excellent conductivity, mechanical strength, and relatively low cost. However, under acidic, high-temperature, and humid environments, stainless steel bipolar plates may be seriously corroded, leading to a sharp change in performance due to the leaching of metal ions [1]. As a result, the design of functional coatings for bipolar plates, as a robust barrier to improve fuel cell performance and stability, has attracted widespread studies in recent years. Such coatings fulfill two primary functions: Firstly, they provide an active area on the electrode surface, facilitating the transport and conversion of reactants [4]. Secondly, coatings can regulate the interface characteristics between the electrode and electrolyte, influencing the efficiency of electron and ion transport. On the consideration of the unique working environment of bipolar plates, materials used for coatings should possess high electrical conductivity, excellent chemical stability, and outstanding corrosion resistance [5,6]. Simultaneously, the preparation process should be cost-effective and scalable to achieve large-scale production in industrial application [7].

Among the candidate materials for anticorrosive and conductive coatings, graphite composed of hexagonal layers of carbon atoms possesses excellent chemical stability and resistance to acid, alkali, and organic solvent corrosion [8]. In a simulated proton exchange membrane fuel cell (PEMFC) environment, the graphite coating placed the anode in a cathodically protected state, reducing corrosion current density and contact resistance, thereby enhancing the corrosion resistance of the copper foil [9]. Liu et al. deposited a silver-doped carbon film on the surface of SS316L stainless steel using nitrogen spray coating technology. The silver-doped carbon film significantly improved the stability and corrosion resistance of SS316L stainless steel while simultaneously reducing contact resistance [10].

Polystyrene, a thermoplastic polymer, is valued for its low weight, high impact resistance, vivid coloration, and excellent chemical corrosion resistance [11]. Compared to resins which require the encapsulation of curing agents, polystyrene is much easier to use in a processing operation. It can be melted and solidified repeatedly within a certain temperature range without undergoing chemical reactions. Corrosion-resistant coatings prepared from polystyrene exhibit chemical stability. According to corrosion principles, polystyrene coatings can reduce film pores through modification or blending with small molecular substances, and a three-dimensional protective structure with higher density was thus established. Such a functional layer provides a shielding effect and its hydrophobicity reinforces the corrosion resistance of the coating [12,13]. In published results, the surface of fabric or filter paper functionalized with SiO_2_ nanoparticles and polystyrene through a one-step impregnation method has been reported. The coating featured a three-dimensional network structure with nanoscale roughness, exhibiting superhydrophobicity and separation functionality [14]. Radwan et al. utilized electrospinning technology to prepare a polystyrene–nickel oxide superhydrophobic nanocomposite coating on the surface of aluminum alloy. The combined action of nickel oxide nanoparticles and polystyrene significantly improved the coating’s hydrophobicity, surface roughness, and thermal stability. Such a treatment led to increased impedance and pore resistance, reduced double-layer capacitance, and effectively inhibited corrosion of the aluminum alloy in saltwater [15]. Zhang et al. prepared a raspberry-like structured polystyrene/silica (PS/SiO_2_) hydrophobic composite coating through electrostatic adsorption and hydrophobic modification with silane. The coating exhibited good mechanical stability and maintained excellent superhydrophobicity after five mechanical friction tests [14].

While significant progress has been made in selecting effective corrosion-resistant materials, achieving dense and highly impermeable coatings on metallic substrates remains a major challenge [16]. Common deposition methods include the following techniques such as electroplating, magnetron sputtering, and physical vapor deposition (PVD) [17]. However, the commercially viable way requires a high-throughput deposition over a large area. The sputtering and evaporation have been performed under a high-vacuum condition. The high degree of ionization, dense film formation, and lower deposition rate have significantly raised the cost of bipolar plates. The experimental approach by way of a solution medium instead of the low-density gas in the vapor phase would definitely show its superiority for faster deposition rates. In addition, the apparatus for a liquid medium is easy to handle and only room temperature is required [18].

**Figure 1:**
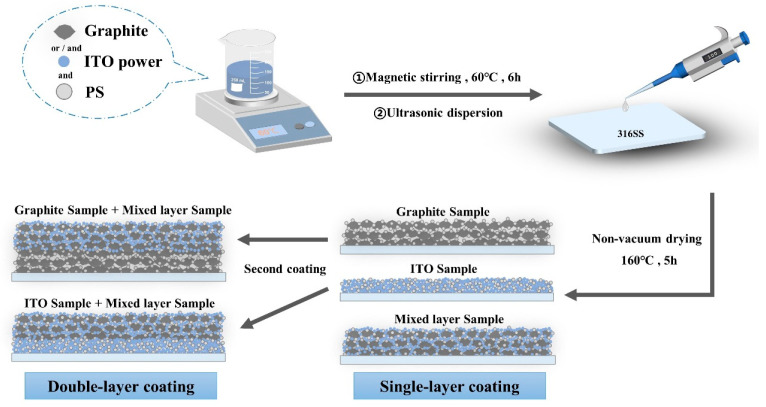
Schematic representation of sample preparation.

In this study, we have developed a cost-effective and mild solution for coating proton exchange membrane fuel cell (PEMFC) bipolar plates. Stainless steel was chosen as the substrate for the coating due to its excellent mechanical properties and corrosion resistance. To achieve coating preparation, we employed a simple and efficient spray or drip coating method to achieve uniformity and consistency of the coating. On this basis, the easily available graphite and indium tin oxide (ITO) powder were employed as conductive fillers to improve the overall conductivity. Simultaneously, a protective shielding layer was formed using polystyrene to efficiently resist corrosive media (Figure 1) [19]. With the aim of strengthening the adhesion and uniformity of the coating, We chose Tween 60 as a surfactant to adjust the formulation, while polystyrene can also be used as a binder [20,21]. As a result, the coated bipolar plates exhibited high electrical conductivity, excellent corrosion resistance, and enhanced hydrophobicity—successfully meeting all technical performance targets established by the U.S. Department of Energy (DOE) guidelines.

## 2. Results and Discussion

### 2.1. Characterization of Coating

**Figure 2:**
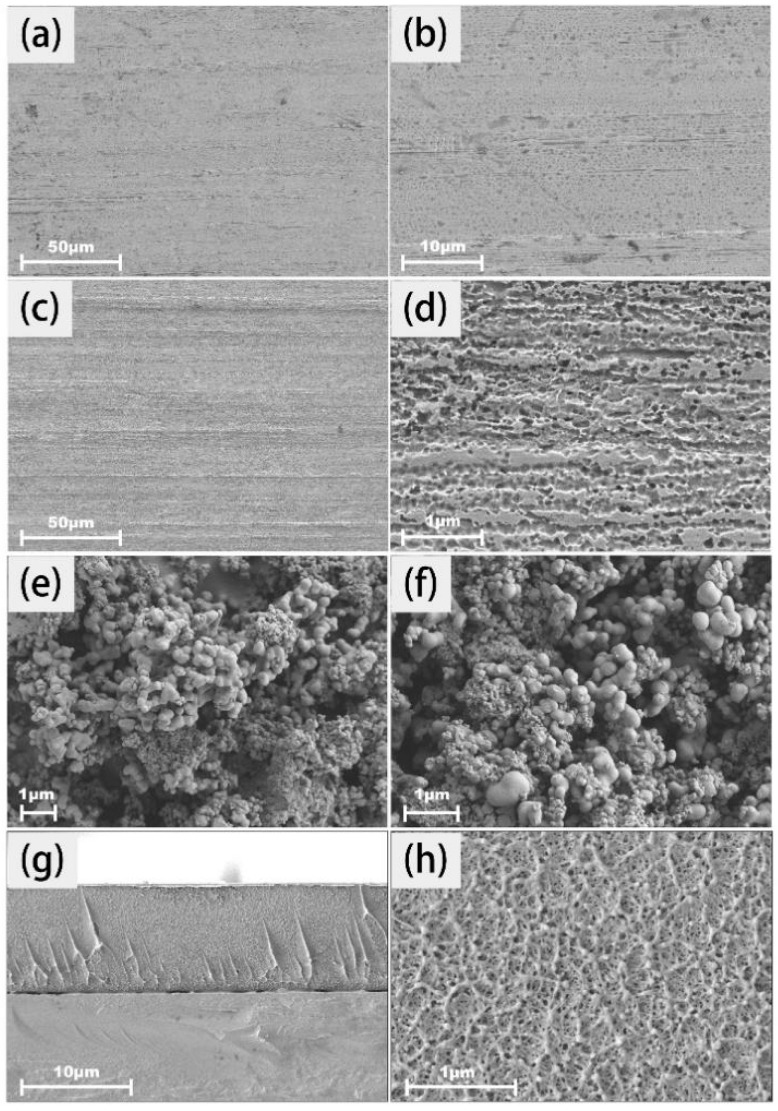
Surface and cross-sectional images captured by SEM: (**a**) Morphology of untreated stainless steel bare plate at 500× magnification; (**b**) morphology of untreated stainless steel bare plate at 2000× magnification; (**c**) morphology of pretreated stainless steel bare plate at 500× magnification; (**d**) morphology of pretreated stainless steel bare plate at 20,000× magnification; (**e**) particle morphology of dissolved polystyrene masterbatch in DMF, dried and magnified 8000×; (**f**) particle morphology of dissolved polystyrene masterbatch in DMF, dried and magnified 15,000×; (**g**) cross-sectional image of polystyrene dissolved in DMF and drop-coated on the plate, magnified 3000×; (**h**) cross-sectional information of the coating in (**g**), magnified 30,000×.

The microscopic information has been given in Figure 2. Several minor scratches and oil stains on the untreated stainless steel bare plate are shown in Figure 2a,b. After ethanol and acid treatment, the stainless steel surface became significantly cleaner and more uniform (Figure 2c,d). Upon observation through a high-magnification image, numerous micropores were clearly identified and such a fact was related to mild etching by dilute hydrochloric acid. The presence of the torturous microstructure contributes to creating a relatively rough surface, allowing the bonding capability between the substrate and the coating [22]. As described in Figure 2e,f, the well-defined polystyrene spherical particles and the average size (200–300 nm) was determined by SEM observation. The cross-sectional image of polystyrene dispersed onto the plate is provided in Figure 2g,h, indicating that the polymeric matrix acted in the encapsulating and interpenetrating role, serving as a compact and reinforcing agent in all directions or dimensions [23].

**Figure 3:**
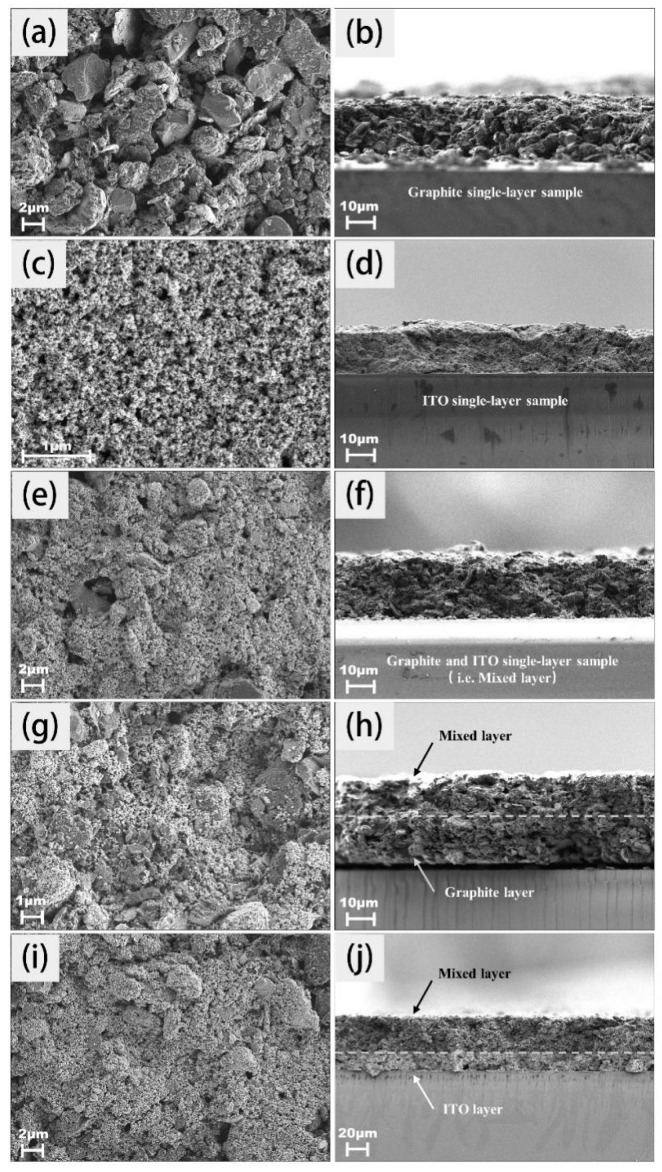
Surface and cross-sectional images captured by SEM: (**a**) Surface morphology of graphite monolayer formula drop-coated on the plate, magnified 3000×; (**b**) cross-sectional image of graphite monolayer formula drop-coated on the plate, magnified 1000×; (**c**) surface morphology of ITO monolayer formula, magnified 20,000×; (**d**) cross-sectional information of ITO monolayer formula, magnified 1000×; (**e**) surface morphology of mixed monolayer formula, magnified 3000×; (**f**) cross-sectional information of mixed monolayer formula, magnified 1000×; (**g**) surface morphology of graphite/mixed double-layer formula, magnified 5000×; (**h**) cross-sectional information of graphite/mixed double-layer formula, magnified 1000×; (**i**) surface morphology of ITO/mixed double-layer formula, magnified 3000×; (**j**) cross-sectional information of ITO/mixed double-layer formula, magnified 300×.

The microscopic image can give effective information on the general structure in the case of a single layer (Figure 3a); the distribution of a few parallel graphite sheet-like structures can be observed, exhibiting the typical layered stacking of graphite crystals. Their rigidity has been controlled by the self-organization into assembled structures and the slight disorder inside and at the edge of the particle would be derived from the large size distribution. In Figure 3b, due to the stacking of large blocks of sheet-like layers, a vacant space within the framework is found in the graphite layers. However, in Figure 3c,d, the particle size of pure ITO powder is ultra-fine and several orders of magnitude smaller than graphite. Its homogenous and regular arrangement has led to almost no significant voids or cracks observed in the cross-sectional image. The full representation of the assembly structure is shown in Figure 3e,f. In the presence of graphite, a large number of ITO conductive particles were incorporated. Such fine powders were uniformly distributed and filled the spaces between the graphite layers. The homogenous morphology was closely related to the rapid growth in conductivity. The polystyrene microspheres established the cross-linked network structure and were considered as a three-dimensional binder, effectively connecting the graphite and ITO particles, creating a tightly bonded composite structure. The integrated structure allows electrons to freely migrate throughout the sample, overcoming the constraints of the layered structure. ITO, as an excellent conductive material, plays a crucial role in providing additional conductive pathways, enhancing conductivity and facilitating electron conduction both laterally and longitudinally in the coating. It should be noted that the analogous densely distributed morphologies are observed in Figure 3g–j. The double-layer coatings exhibited the increasing size in thickness in comparison with the single-layer samples. Such spontaneous growth along the vertical direction contributed to better corrosion resistance by providing powerful barrier effects against acidic media [24].

**Figure 4:**
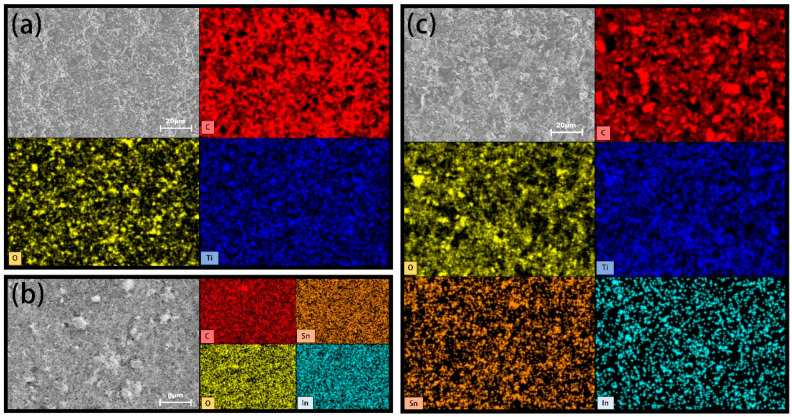
(**a**) Elemental distribution map of the single-layer graphite formula; (**b**) elemental distribution map of the single-layer ITO formula; (**c**) elemental distribution map of the single-layer mixed formula.

**Figure 5:**
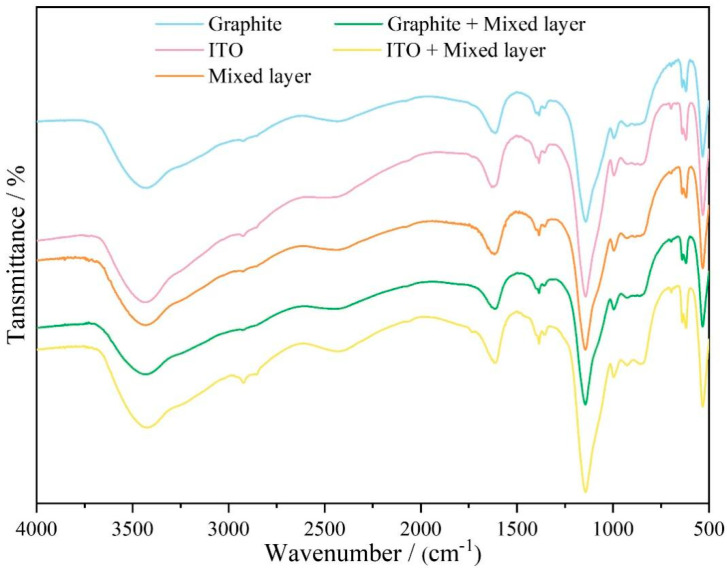
Fourier-transform infrared (FTIR) spectra of different samples.

The chemical composition and elemental distribution are demonstrated in Figure 4. The presence of carbon, indium, and tin have been verified and the proportion of titanium would be correlated by the minor element in stainless steel 316. The fine interpenetrating microstructures with extensive surfaces could assemble tortuous pathways to improve barrier performance. Organic components, such as polystyrene and Tween 60, are embedded within the coatings, as confirmed by FTIR analysis in Figure 5 and Table S1. The inclusion of organic segments would be beneficial for inhibiting the dispersion of corrosion species. A broad peak in the range of 3463–3426 cm^−1^ corresponds to the hydroxyl group stretching vibration peak adsorbed by the coating molecules, indicating the presence of a large number of hydroxyl functional groups onto the surface [25]. The band observed at 2925–2853 cm^−1^ has been ascribed to the C-H stretching vibration of the main chain of polystyrene. In the position of 1620–1610 cm^−1^, the C=C stretching vibration of the aromatic ring in the benzene ring and the C=O stretching vibration in the Tween material are found. The characteristic bands in the range of 1410–1350 cm^−1^ would be attributed to C-O, C-H bending vibrations, and the C-O-C stretching vibration of esters. The signal at 1140 cm^−1^ has been contributed by the symmetric stretching vibration of the ester group C-O-C. The weak bands in the range of 995–840 cm^−1^ are assigned to the C-H in-plane bending vibration. The series peaks in the range of 698–530 cm^−1^ are derived from various vibration modes of the aromatic ring out-of-plane skeleton and the out-of-plane bending vibration of C-H [25].

**Figure 6:**
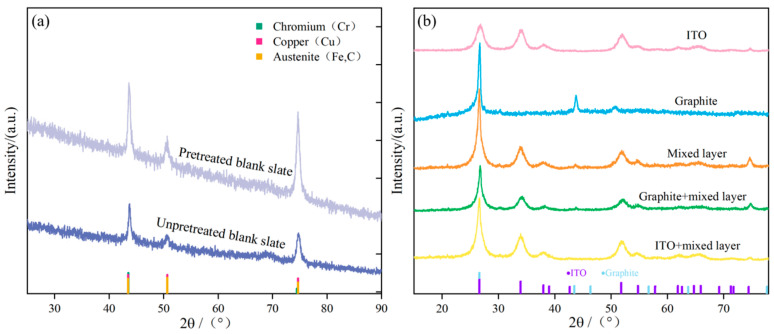
X-ray diffraction (XRD) patterns of different samples: (**a**) The XRD spectra of untreated stainless steel bare board and stainless steel bare board after pretreatment; (**b**) from top to bottom, the XRD patterns of single-layer coatings of ITO, graphite, mixed layer, and composite double-layer coatings of graphite/mixed layer and ITO/mixed layer.

The X-ray diffraction patterns of the untreated and treated stainless steel substrates are given in Figure 6a. It can be observed that the substrate’s composition and structure remain unchanged regardless of pretreatment. Characteristic diffraction peaks at 2θ = 43.51°, 50.67°, and 74.49° corresponded to the (111), (200), and (220) crystal planes of chromium, while signals at 2θ = 43.47°, 50.67°, and 74.68° were assigned to the (111), (200), and (220) crystal planes of copper. Similarly, peaks at 2θ = 43.47°, 50.67°, and 74.68° were relevant to the (111), (200), and (220) crystal planes of stainless steel iron. The peak positions for these three substances are in close proximity. The results showed that the ultrasonic treatment by alcohol and acid for around 30 min (with washing process) yields a more intensive diffraction pattern and such a feature supported the improvement in crystallinity of the stainless steel substrate. After the samples were subjected to a cleaning process to remove the impurities on the surface, the number of crystalline planes oriented in given directions would increase, and the possibility of lattice distortion or crystal defects induced by contaminants could be avoided.

Upon the addition of graphite and ITO powder, all five samples exhibit analogous diffraction peaks (Figure 6b). Graphite possesses peaks at 2θ = 26.611° and 43.453°, corresponding to the (111) and (010) crystal planes. Samples containing ITO powder exhibit distinct peaks at 2θ = 26.611°, 33.893°, 37.949°, 38.968°, and 51.780°, corresponding to the (110), (101), (200), (111), and (211) crystal planes. These curves predominantly indicate the presence of tin dioxide (SnO_2_) in the ITO powder, suggesting the presence of the main functional component. The other raw material in the ITO powder, indium oxide (In_2_O_3_), is found in a smaller quantity, which has not been individually marked in this figure.

### 2.2. Characterization of Electrochemical Properties of Coatings

**Figure 7:**
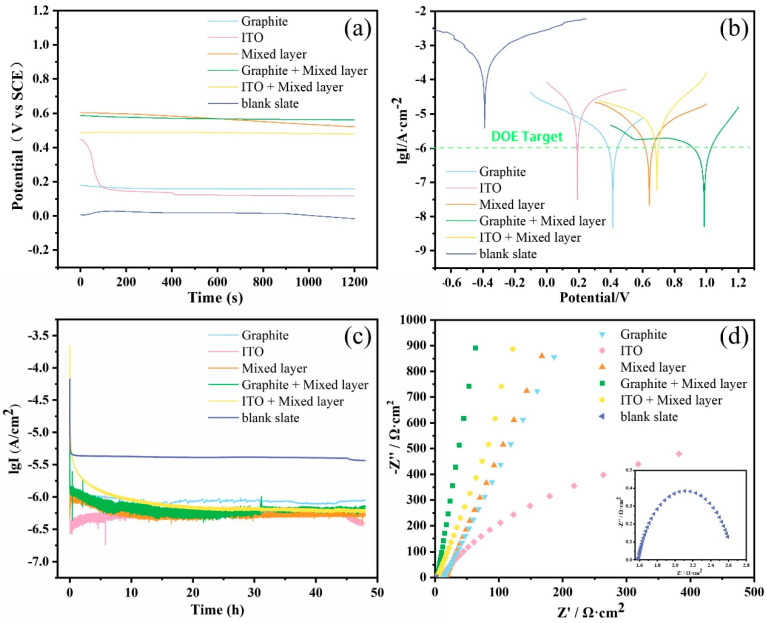
The results of four electrochemical tests for different samples: (**a**) The 20-minute open-circuit potential (OCP) test; (**b**) potentiodynamic polarization test; (**c**) 48-hour potentiostatic test; (**d**) electrochemical impedance spectroscopy (EIS) test.

Coating performance and corrosion resistance were evaluated by monitoring open-circuit potential (OCP) over time [26]. Generally, a lower OCP value verifies more severe corrosion, while a higher OCP for coated or passivated surfaces suggests better corrosion resistance [27]. Additionally, OCP can reflect the corrosion intensity and rate: a faster decline tendency is related to a higher corrosion rate. As shown in Figure 7a, stainless steel coated with the formulated components exhibits a higher OCP compared to the bare metal, and the OCP of the double-layer composite coating is higher than that of the single-layer coating, indicating that the presence and thickness of the coating are crucial factors for stainless steel corrosion resistance. However, with increasing immersion time, the OCP of stainless steel samples coated with the mixed formulation gradually decreases, suggesting the protective performance of the coating on the metal has become worse. Different coating systems would be variable to reach a relatively stable OCP during immersion, mainly due to the resistance difference in ion penetration. Coatings with a better resistance effect require an extension of time to achieve a stable OCP. Therefore, the time to reach a stable OCP value can be used to assess the coating’s protective performance [28]. It is evident from the data that, although all samples show a slight decrease in OCP, the decline in the composite coating is insignificant, indicating that it requires a longer time to achieve a stable OCP and possesses superior corrosion resistance. Furthermore, specific numerical values demonstrate that all electrode samples covered with coatings maintain an average current density below 1 μA/cm^2^ during long-term testing and the values could meet the standard of the U.S. Department of Energy.

The potentiodynamic polarization behavior of different formulation-coated samples under a simulated proton exchange membrane fuel cell (PEMFC) environment has been studied in Figure 7b. The bare SS316L sample exhibits no significant passivation state in the simulated anodic and cathodic environments, indicating the passivation film has been removed on the coated sample surface after pretreatment. However, it is evident that both single-layer and double-layer coated samples significantly improve the corrosion potential and reduce the corrosion current density compared to the bare plate. Data fitting based on the information in the graph yields the current density values presented in Table 1. The current densities for the single-layer coatings of graphite, indium tin oxide (ITO), and the mixed layer are calculated to be 0.59 μA/cm^2^, 9.52 μA/cm^2^, and 0.76 μA/cm^2^, respectively. The corresponding double-layer coatings exhibit current densities of 0.52 μA/cm^2^ and 1.89 μA/cm^2^, and intrinsically meet the corrosion current density requirements raised by the U.S. Department of Energy. The higher corrosion current density observed in the ITO-containing samples may be attributed to the unique conductivity of ITO powder, leading to increased activity in the electrochemical condition and the generation of strong polarization active sites on the coating surface. However, the slight increase in value has a trivial effect on corrosion protection. Moreover, the corrosion potential measured in this study is larger than most reported corrosion potentials for bipolar plate coatings, indicating that the functional coating provides more effective cathodic protection in the severe acidic environment of PEMFC anodes [29]. The Protection Effectiveness (P.E.) is a common method for evaluating sample corrosion resistance [30], and it can be calculated from the measured I_corr_ values using the formula:

$$P.E.=\frac{\left(I_{corr,bare}-I_{corr,coated}\right)}{I_{corr,bare}}\times 100$$

To assess the durability of the coatings in a simulated working environment, a 48 h potentiostatic polarization study was conducted. Figure 7c illustrates the recorded current density variation over time. Due to the presence of high potential in the simulated cathodic environment, bipolar plates are more prone to corrosion compared to the anodic condition. Coatings (above the standard of the U.S. Department of Energy) suitable for application in the simulated cathodic process could also be effective under simulated anodic conditions [31]. Accordingly, the long-term stability of the coatings in a simulated cathodic environment (+0.6 V) has been investigated. In Figure 7c, it can be observed that the current density rapidly decreases at the initial stage, followed by minor fluctuations. Throughout the extended testing period, the average corrosion current density of both single-layer and composite coatings remains lower than the U.S. Department of Energy’s requirement of 1 μA/cm^2^. Consequently, the coatings exhibit excellent corrosion resistance, and the bipolar plates can be safely protected in terms of the assembly of the above films.

**Figure 8:**
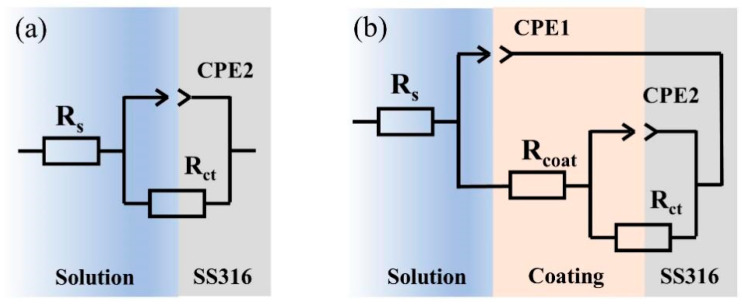
(**a**) Equivalent circuit model for the impedance test of the bare plate; (**b**) equivalent circuit model for the impedance test of the coated sample.

Electrochemical impedance spectroscopy (EIS) results were fitted using the equivalent circuit model shown in Figure 8, where R_s_ represents solution resistance, R_coat_ is termed as coating resistance, CPE1 corresponds to the constant phase element related to the coating, R_ct_ is defined as charge transfer resistance, and CPE2 represents the constant phase element associated with the double layer. The fitting results for R_ct_ are illustrated in Figure 7d and summarized in Table S2. It is evident that the composite coating graphite/mixed layer demonstrates the highest charge transfer resistance (R_ct_). A higher R_ct_ value is equal to a stronger corrosion resistance. In the Nyquist plot, the diameter of the capacitive arc provides insights into the coating’s corrosion resistance. A larger diameter suggests a more robust corrosion resistance of the coating. The capacitive arc diameter of the graphite/mixed layer surpasses the other coating samples; the value is several orders of magnitude higher than the bare plate. The results reveal its superior corrosion resistance, followed by the composite coating ITO/mixed layer samples and single-layer samples.

**Figure 9:**
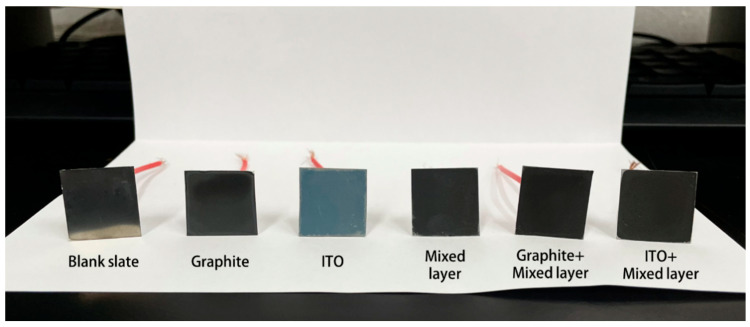
Appearance of coating after electrochemical test.

The electrochemical analysis data have supported that the composite coating, compared to the single-layer coating, possesses a reinforcing coating and superior resistance to corrosion medium invasion. The thickness of the coating is closely related to its anticorrosion feature. The graphite, due to its layered structure, effectively blocks more corrosive media from lateral erosion. During the drying process of the second layer coating, the upper layer’s formulation can compensate for the defects generated when applying the lower layer, and the risk of pitting corrosion in a single-layer coating can be avoided. Meanwhile, the ITO powder, serving as a conductive filler, effectively fills the empty space between the graphite layers and the small microsphere structure of the polymer. The internal porous structures have been packed and the speed at which the corrosive medium spreads to the substrate has been lowered. All samples after treatment are studied in Figure 9; the lowest chemical stability has been found in the sample of the bare plate. However, the functional coatings involved in this study could inhibit the penetration of corrosive species.

### 2.3. Characterization of Coating Surface Contact Resistance

**Figure 10:**
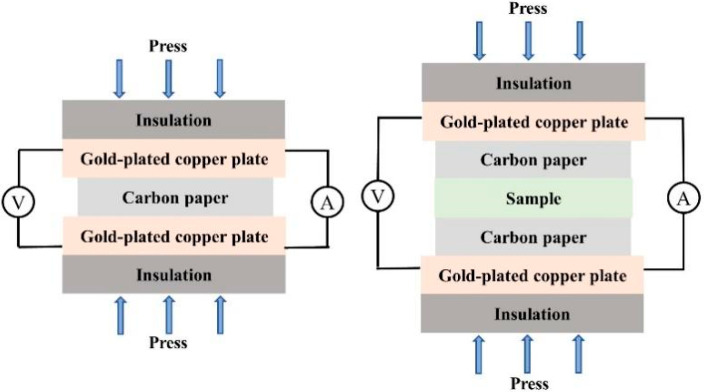
Test model of interface contact resistance.

Interfacial contact resistance (ICR) is critical for bipolar plate performance, as high ICR reduces PEMFC efficiency and lifespan [32]. The numerical values of surface contact resistance for different coated plates, carbon papers, pretreated plates, and untreated plates were investigated under different compaction pressures, as illustrated in the contact resistance measurement model [33] shown in Figure 10. Typically, if a coating exhibits good conductivity, lower ICR values can be achieved. As shown in Figure S1, the ICR values for both bare plates and coated samples rapidly decrease with increasing pressure initially, then a slow decline around 0.8 MPa is found, and finally, they tend to stabilize when the pressure reaches a certain value. The actual contact area between interfaces increases with the compaction pressure, transforming from an initially nonuniform surface with rough structures to a gradually flattened and compressed state, leading to an increase in conductivity [34]. Specific data from Table 2 indicate that the ITO coating formulation and two composite coating formulations could meet DOE standards at 1.65 MPa. The data proved the excellent conductivity of the coating under this pressure, attributed to the filling and synergistic action between the conductive powder and graphite. With the assistance of higher pressure, the conductive powder effectively fills the gaps between graphite layers laterally, acting as a cover and channel. In the vertical direction, the conductive powder inherently reinforced the connection between graphite layers, filling small voids in the internal structure, allowing electrons and current to flow smoothly. A large conductive network has been formed and the poor conductivity of the polymer filler has been compensated. The higher contact resistance of untreated bare plates can be attributed to the presence of an oxide layer on the surface. The passivation film significantly increases contact resistance. It is the reason why we have to wash using acid treatment in the process of the pretreatment operation.

### 2.4. Characterization of Coating Contact Angle

**Figure 11:**
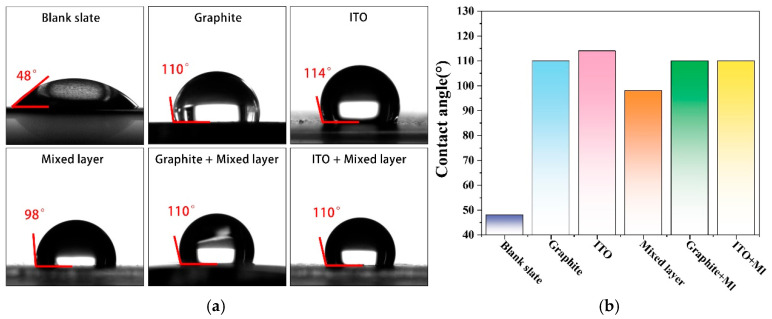
(**a**) Contact angle pictures of dripping 3 μL water droplets on different sample surfaces; (**b**) columnar comparison of contact angle data.

Under the influence of intermolecular forces, water tends to form a sphere to minimize surface tension. If the coating surface exhibits micro-nanostructures, it creates numerous small vacant pores. Due to the dimensions and size of these structures, water droplets, being relatively large in comparison, cannot penetrate them. This fact results in a state where water droplets are supported on the coating surface without being absorbed [35]. In other words, suppose the surface’s uneven dimensions are small enough, water droplets could not wet the sample, displaying a lotus effect. The wettability of the coating surface measured by a contact angle goniometer is given in Figure 11a. All coating formulations can enhance the hydrophobicity compared to the inherent hydrophilicity of the bare plate. Overall, they exhibit hydrophobic characteristics, with contact angles greater than 90° and around 110°, as visually compared in Figure 11b. After drying, coatings with finer surface roughness exhibit better hydrophobicity. Single-layer samples made from conductive fillers can assemble more delicate microstructures and control water penetration. Composite coatings, benefiting from the synergistic effect of graphite and conductive fillers, also lead to similar results. In general, they possess hydrophobic effects, and the operational conditions of bipolar plates have been considered. The hybrid layer serves water-repelling and corrosion prevention purposes, consistent with previous experimental data.

## 3. Experimental Section

### 3.1. Materials

Stainless steel 316 plates were supplied by Dongguan Xinchengyu Metal Products Co., Ltd. (Xinchengyu, Dongguan, China). Graphite powder was provided by Southern Graphite (Southern Graphite, Chenzhou, China) New Material Technology Co., Ltd. Indium tin oxide (ITO) powder (20 nm) was purchased from Star-Research New Materials Co., Ltd. (Star research, Suzhou, China). Anhydrous ethanol was provided by Tianjin Zhiyuan Chemical Reagent Co., Ltd. (Zhiyuan, Tianjin, China). Polystyrene (PS, general type I) and Tween 60 (T60) were both obtained from Aladdin Company (Aladdin, Shanghai, China). All reagents and solvents were purchased from commercial suppliers and used without any further treatment. Deionized water was provided by the university after undergoing centralized processing.

### 3.2. Preparation of Stainless Steel Substrate

The original material consists of 0.1 mm thick 316SS sheets (Xinchengyu, Dongguan, China), which were later processed in the laboratory by cutting them into small square pieces (1 cm × 1 cm) for subsequent use. The samples were subjected to a 15 min ultrasonic de-oiling process using anhydrous ethanol, followed by immersion in acid solution (HCl 40 g/L) and ultrasonic agitation for 15 min to remove surface oxides. Depending on the situation, the acid treatment time was appropriately extended for surface etching to increase roughness, enhancing the adhesion of subsequent coating formulations [36,37]. After each cleaning step, the samples were rinsed three times with both regular water and laboratory-grade deionized water to eliminate residual reagents [38].

### 3.3. Preparation of Coating Formulations

An amount of 4 g of polystyrene was dissolved in 0.2 L of N,N-dimethylformamide, and 6.55 g of Tween 60 was dissolved in 0.2 L of deionized water. The solutions were thoroughly mixed by magnetic stirring at 50 degrees Celsius for 6 h to ensure complete and uniform dissolution of the solutes, creating large batches of prepared solutions. Additionally, polystyrene sample boards were introduced as a supplementary experimental group for electron microscope morphology. The specific sample quantities are detailed in Table 3. After mixing all formulations, they were magnetically stirred at 60 degrees Celsius for another 6 h. Subsequently, each formulation was sonicated for 1 h and immediately spread onto the surface of 316SS, with a controlled application volume of 100 μL per sample. The coated samples were then placed in an oven and dried at 160 degrees Celsius for 5 h. Afterward, they were allowed to cool to room temperature. The manufacturing process is illustrated in Figure 1.

### 3.4. Sample Encapsulation

The backside of the prepared samples is connected to copper wires, tightly affixed with conductive adhesive. The reverse side is completely sealed using 704 L silicone rubber, exposing only the area to be tested with the coating (exposed area of 1 cm^2^). After curing at room temperature for half an hour, the samples are ready for electrochemical testing.

### 3.5. Characterization

High-resolution surface morphology and cross-sectional images of the coatings were captured using a Scanning Electron Microscope with a beam acceleration voltage of 2.0 kV (SEM, Zeiss Gemini 500, Oberkochen, Germany). The intrinsic principle of such characterization relies on the electron gun in the equipment producing a beam of electrons; the sample has been scanned and signals are achieved. The sample was air-pumped to remove impurities from the surface and pasted on the base of the sample stage with conductive tape. The instrument was loaded to a different magnification and the observation images were recorded. The stainless steel bare plates in the absence or presence of pretreatment were explored by SEM images via 500× or 2000× magnification. Polystyrene particles or dispersion onto the plates were explored by SEM images via different magnification. The graphite monolayer, ITO monolayer, or mixed layers were investigated by SEM images via different magnification. Additionally, an Energy-Dispersive X-ray Spectrometer (EDS) (Quantax 400, Bruker, Germany)was employed to analyze the elemental composition and relative content of the sample surface, providing elemental distribution maps.

Chemical bond information within the coatings was obtained using a Fourier-Transform Infrared Spectrometer (FTIR) to confirm the functional groups and chemical composition of the coatings. The phase composition of the coating materials was determined using X-ray Diffraction (XRD). Corrosion behavior in a simulated proton exchange membrane fuel cell (PEMFC) environment (0.5 mol/L H_2_SO_4_, 10 ppm Cl^−^, 30 ppm F^−^, 70 °C constant temperature water bath) was compared between uncoated stainless steel plates and coated samples with the mentioned formulations. Electrochemical tests, including Open Circuit Potential (E_ocp_), Electrochemical Impedance Spectroscopy (EIS), and Tafel dynamic polarization, were conducted using a CHI660E electrochemical workstation. A saturated calomel electrode served as the reference electrode, and a 1 cm^2^ platinum electrode was used as the counter electrode. The samples underwent a half-hour open circuit potential stability test before impedance testing with the IMP-AC impedance program (2.0 edition). A 5 mV AC signal was applied, and the amplitude was scanned from 100 kHz to 0.01 Hz. Tafel dynamic polarization measurements were performed within a voltage range of ±500 mV at a scan rate of 1 mV/s [39,40]. Corrosion resistance performance was tested under a constant potential in a simulated cathodic environment (+0.6 V) for 48 h. Contact resistance under ambient temperature and pressure was measured using a universal testing machine with a current source and multimeter. Contact angle (CA) values were obtained from five different positions on each sample using a JC2000D1 Optical Contact Angle Measurement Instrument. The images of the droplets were acquired using the instrument’s built-in software (1.0 edition). All experiments were repeated three times, and data with good repeatability were selected to ensure accuracy [41].

## 4. Conclusions

In this study, we developed a simple and cost-effective drip or spray coating method to prepare single-layer and double-layer composite coatings for PEMFC bipolar plates. Through coordinated regulation of dual conductive species and a single plastic component, along with the addition of a surfactant to optimize coating surface uniformity, we achieved coatings with excellent smoothness and high stability, meeting the requirements for both electrochemical performance and contact resistance. The layered graphite and the microsphere structure of polystyrene provided a lateral physical barrier coverage, while the conductive powder, due to its small particle size, smoothly filled the gaps between graphite and polystyrene microspheres, reducing internal voids. Such a change resulted in a vertically and laterally interconnected conductive network, and the stability to resist corrosive media has been strengthened. Scanning electron microscopy images revealed effective particle filling and coverage effects in the internal structure. Infrared absorption indicated that polystyrene and the added Tween-type emulsifier were included in the composition components, ensuring coating adhesion and uniformity. Extensive electrochemical analysis demonstrated a significant enhancement in corrosion resistance for the coated samples, with the average current density below 1 μA/cm^2^. Tafel polarization results for graphite coating, ITO coating, single-layer coatings with mixed-layer formulations, and their corresponding double-layer coatings were 0.59 μA/cm^2^, 9.52 μA/cm^2^, 0.76 μA/cm^2^, 0.52 μA/cm^2^, and 1.89 μA/cm^2^. In the contact resistance test, the ITO coating and two double-layer composite coatings could meet the principle of DOE standards at 1.65 MPa. Interface contact resistance testing indicated that the oxide film on the exposed stainless steel surface was a critical factor in increasing interface contact resistance. The selected conductive filler effectively compensated for the low conductivity of polystyrene, not only allowing the overall conductivity of the bipolar plates but also providing a certain degree of corrosion resistance. Contact angle tests demonstrated that the prepared coatings exhibited excellent hydrophobicity. Overall, the performance of graphite/mixed layer and ITO/mixed layer would be superior, followed by mixed layer, graphite, and ITO single-layer coatings. This innovative approach offers a low-cost, easily scalable, and durable coating solution for fuel cell bipolar plates, providing new opportunities for cost reduction and enhanced protection against corrosive environments.

## Figures and Tables

**Table 1:** The fitting data after potentiodynamic polarization test of different samples.

Sample	E_corr_ (V)	Icorr (μA/cm^2^)	P.E. (%)
Graphite	0.42	0.59	99.867
ITO	0.16	9.52	97.862
Mixed layer	0.64	0.76	99.829
Graphite + Mixed layer	0.98	0.52	99.883
ITO + Mixed layer	0.69	1.89	99.575
Blank slate	−0.38	445.24	—

**Table 2:** Original data of interface contact resistance.

Interfacial Contact Resistance (mΩ·cm^2^)								
Force (N/cm^2^)	Force (Mpa)	Graphite	ITO	Mixed Layer	Graphite+ Mixed Layer	ITO+ Mixed Layer	Unpretreated Blank Slate	Pretreated Blank Slate
15	0.15	1948	1668	1188	1868	1508	2748	328
30	0.3	1450	1370	1010	1430	1250	2190	290
45	0.45	1140	980	980	1260	1140	1740	260
60	0.6	806.8	746.8	866.8	1146.8	946.8	1326.8	206.8
75	0.75	509.4	309.4	569.4	949.4	869.4	1149.4	109.4
90	0.9	497.6	297.6	477.6	817.6	457.6	1017.6	97.6
105	1.05	486.8	286.8	426.8	386.8	406.8	746.8	66.8
120	1.2	474.6	214.6	374.6	294.6	314.6	674.6	54.6
135	1.35	361.8	161.8	221.8	241.8	201.8	561.8	41.8
150	1.5	136.4	16.4	56.4	16.4	16.4	536.4	36.4
165	1.65	85.4	5.4	45.4	3.4	1.4	505.4	5.4

**Table 3:** Parameters of raw materials for different samples.

Coating	Graphite Powder (g)	ITO Powder (g)	Polystyrene (in DMF/mL)	Tween 60 (in Deionized Water/mL)
Single-layer sample				
Graphite	0.5	—	3	2
ITO	—	0.5	3	2
Mixed layer (Ml)	0.3	0.2	3	2
Double-layer sample				
Graphite/Ml	0.5 + 0.3	—	3 + 3	2 + 2
ITO/Ml	—	0.5 + 0.2	3 + 3	2 + 2

## Data Availability

The data can be shared upon request.

## Supplementary Materials

The following supporting information can be downloaded at: https://www.mdpi.com/article/10.3390/molecules30122543/s1, Figure S1. The changing trend of interface contact resistance of different samples and its curve magnification at 1.2~1.65 Mpa. Table S1. Infrared vibration modes of polystyrene and Tween contained in different samples. Table S2. The fitting results of electrochemical impedance measurements of different samples obtained by Zview software (1.0 edition).
