# Supplementary material for: A Non-Vacuum Coating Process That Fully Achieves Technical Goals of Bipolar Plates via Synergistic Control of Multiple Layer-by-Layer Strategy
Source: Molecules. 2025 Jun 11;30(12):2543. doi: 10.3390/molecules30122543 (PMC12196416; doi:10.3390/molecules30122543)
Supplement: Supplementary file 1 [file molecules-30-02543-s001.zip › molecules-3692261-supplementary.pdf]

Supporting information for

A Non-Vacuum Coating Process That Fully Achieves Technical Goals of Bipolar Plates via Synergistic Control of Multiple Layer-by-Layer Strategy

Qiaoling Liu †, Xiaole Chen †, Menghan Wu, Weihao Wang, Yinru Lin, Zilong Chen,

Shuhan Yang, Yuhui Zheng * and Qianming Wang *

Key Laboratory of Analytical Chemistry for Biomedicine, School of Chemistry, Guangzhou South China Normal University, Guangzhou 510006, China

* Correspondence: yhzheng78@scnu.edu.cn (Y.Z.); qmwang@scnu.edu.cn (Q.W.)

† These authors contributed equally to this work.

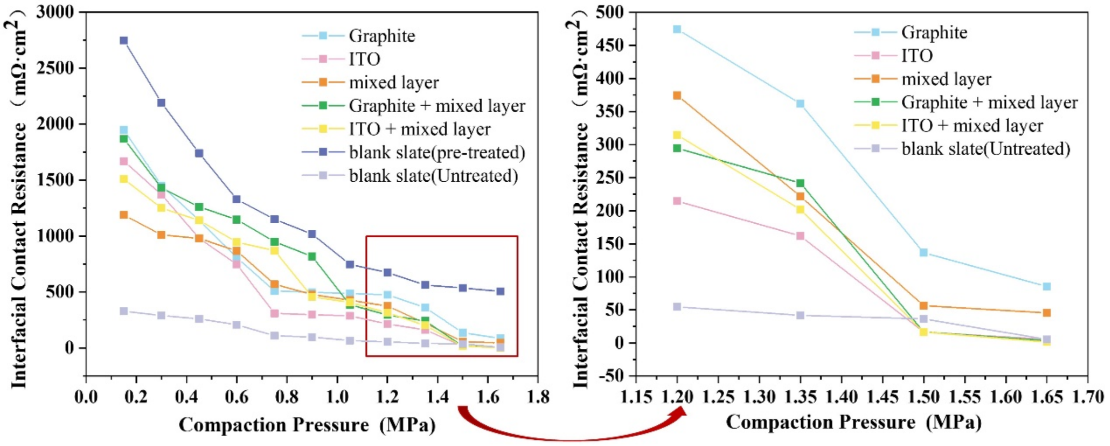

Figure S1. The changing trend of interface contact resistance of different samples and its curve magnification at 1.2~1.65Mpa.

Table S1. Infrared vibration modes of polystyrene and Tween contained in different samples.

**Polystyrene + Tween 60**	
**Wavelength (cm^{-1})**	**Mode**
3426-3463	O–H hydroxyl groups
2925-2853	C-H stretching
1620-1610	C=O stretching
1410-1350	C-O stretching、C-H bending vibration、C-O-C stretching
1140	C-O-C symmetric stretching
995-840	C-H in-plane bending
698-530	Several vibration modes of the outer skeleton of aromatic rings and C-H out-of-plane bending

Table S2. The fitting results of electrochemical impedance measurements of different samples obtained by Zview software.

Sample	R_s(Ω·cm^2)	R_{coat}(Ω·cm^2)	CPE1 (Ω·cm^{-2}·S^{-n}×10^{-6})	n_1	R_{ct}(Ω·cm^2)	CPE2 (Ω·cm^{-2}·S^{-n})	n_2
Graphite	0.70618	3.095	0.019575	0.9319	2.238×10^{9}	0.036602	0.87381
ITO	0.92728	1.732	0.066922	0.80387	384	0.0051006	0.83159
Mixed layer	0.11119	0.85862	0.010355	1.029	3.006×10^{10}	0.063266	0.95745
Graphite+ Mixed layer	0.27135	3.739	0.65034	0.98908	1.374×10^{11}	0.032091	0.88897
ITO+ Mixed layer	0.75403	0.44717	3.6105	0.89967	3.633×10^{10}	0.087811	0.90244
Blank slate	0.39658	—	—	—	0.26623	4.771	0.79701
